# CD38 – Negative Anaplastic Plasma Cell Myeloma: A Rare Case Report

**DOI:** 10.7759/cureus.20909

**Published:** 2022-01-03

**Authors:** An Thi Vinh Do, Le Lan Anh

**Affiliations:** 1 Internal Medicine • Hematology and Oncology, Bach Mai Hospital, Hanoi, VNM; 2 Internal Medicine • Hematology, Bach Mai Hospital, Hanoi, VNM

**Keywords:** anaplastic, flow cytometry, cd38 negative, cd38, multiple myeloma

## Abstract

CD38 is a glycoprotein that is highly and uniformly expressed in plasma cells in multiple myeloma. A panel of CD38 and CD138/CD19/CD45/CD56/CD117 markers is considered the immunophenotypic diagnosis of plasma cell myeloma. Expression of the CD38 marker may fade or weaken compared with the CD138 marker in plasma cells after chemotherapy treatment. Herein we present a rare case of CD38-negative multiple myeloma that was initially misdiagnosed as acute leukemia.

## Introduction

According to WHO 2016, multiple myeloma is a hematologic malignancy, usually in the bone marrow with an increase of more than 10% of plasma cells, with morphological identifications, immunological markers, and chromosomal abnormalities. Peripheral blood has abnormal immunoelectrophoresis, rouleaux formation, high level of Lactate dehydrogenase (LDH) with lytic bone lesions on MRI [1]. Anaplastic plasma cell myeloma is a rare, refractory subtype of multiple myeloma and has a poor prognosis. However, abnormal morphology and asynchronous immunophenotypic expression of plasma cells can be seen in newly diagnosed cases of multiple myeloma and often cause the inaccuracy of initial diagnosis [2].

## Case presentation

A 46-year-old female patient with unsignificant past medical history presented with fatigue and dyspnea for the previous three months. The physical examination was unremarkable. ENT endoscopy showed a mass in the nose, histopathology of the biopsy piece of this tumor revealed nasopharyngeal cancer. The patient underwent Intensity-modulated radiation therapy (IMRT) (dose 42Gy/21fx) and chemotherapy with a Cisplatin regimen at a local hospital. After six days of radiation therapy, the patient appeared to have a lot of nosebleeds and severe back pain.

**Figure 1:**
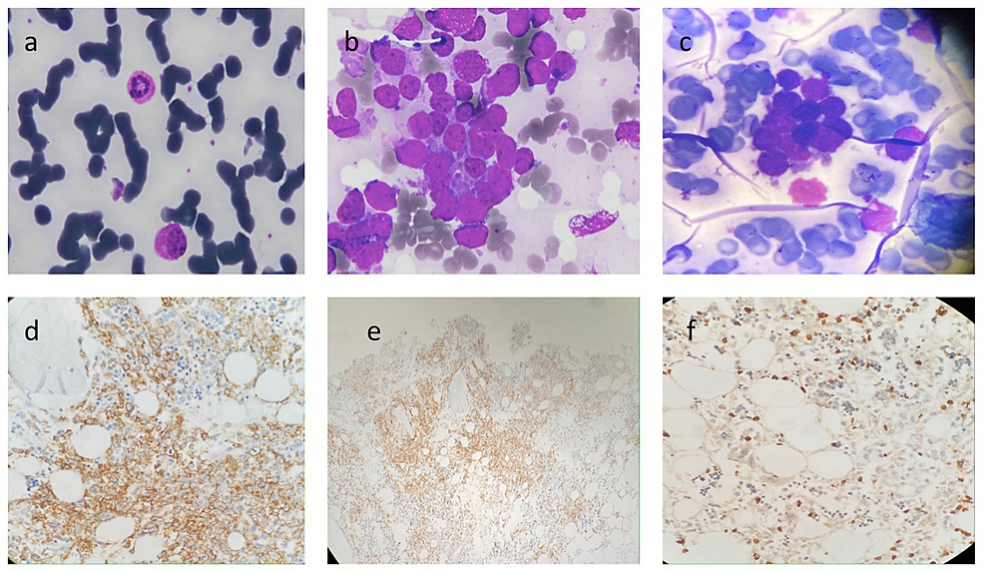
Peripheral smear, bone marrow aspiration and biopsy (a) Peripheral smear: rouleaux formation and large atypical cells. (b) Bone marrow smears imprints show large atypical cells with marked nuclear convolutions. (c) negative for Sudan black B staining. (d) Immunohistochemistry on marrow biopsy: positive CD138, (e) positive CD56, (f) positive Ki67

Complete hemogram showed Hb = 8.6 g/dl, Platelet Count (PLT) = 19 x 10^9^/L, WBC = 0.7 x 10^9^/L and a differential count of N:66 L:23 M:5. The peripheral blood smear revealed a leuco-erythroblastic picture with thrombocytopenia, rouleaux formation, and large atypical cells. Bone marrow smears showed 72% large atypical cells with marked nuclear convolutions that were negative for all cytochemical staining markers (Figure 1). A preliminary diagnosis of acute leukemia/ nasopharyngeal cancer was made, and further investigations were performed. Biochemical markers revealed high levels of LDH (1842 IU/ml) while total serum protein was 6.6 g/dl, serum albumin was 3.39 g/dl with an A: G ratio of 1.06: 1. Serum calcium was 9.12 mg/dl, renal and liver function tests were normal. Quantification of heavy chain immunoglobulins was normal, with kappa/lambda ratio: 12,9:24,7 (0,52:1). HIV, HBV, HCV viral serology were all negative. Ultrasound examination revealed no lymphadenopathy or organomegaly. Magnetic resonance imaging (MRI) showed multiple lytic bone lesions on the lumbar spine L1 - L5 and bilateral sacrum and pelvis, which highly suggested plasma cell myeloma.

**Figure 2:**
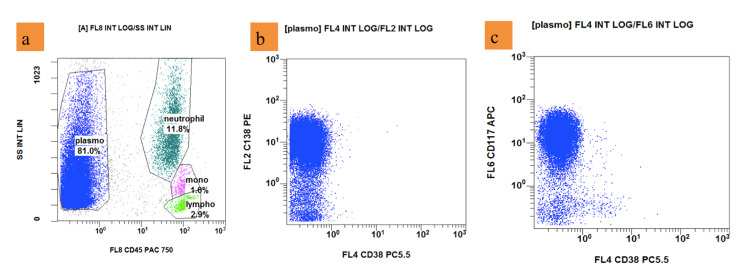
Flow cytometry (a) Plasma cells (blue dots) account for 81.0%; are negative for CD45; (b): positive for CD138, negative for CD38; (c): positive for CD117 (Navios EX - Beckman Coulter)

**Figure 3:**
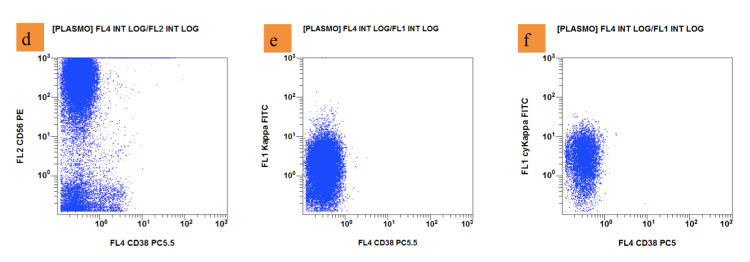
Flow cytometry (d): Plasma cells (blue dots) are positive for CD56; (e) positive for kappa; (f) positive for Cytoplasmic kappa and negative for CD38 (Navios EX - Beckman Coulter)

**Figure 4:**
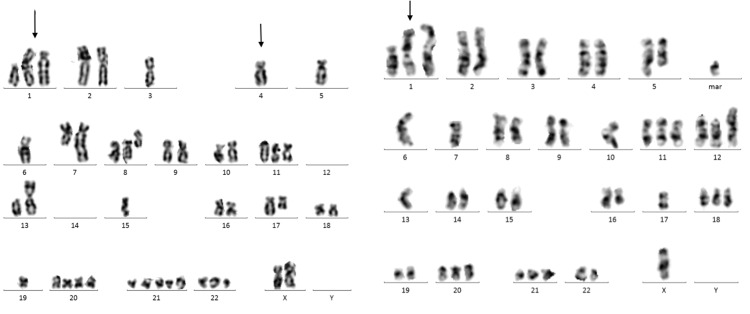
Karyotype abnormalities gain (1q), del (4q)

The initial panel of flowcytometric immunophenotyping on bone marrow was negative for CD45, CD34, TdT, HLA-DR, CD19, CyCD3, CD79a, MPO, CD71, CD235, CD41, CD61, and CD38 markers. An extended panel consisting of CD138, CD56, CD117 was applied and came out positive along with a kappa light chain (Figures 2, 3). Hypercellular bone marrow biopsy sections revealed near-total replacement of undifferentiated large atypical cells that were strongly positive for CD138, CD56, Ki67 but negative for CD38 (Figure 1). Cytogenetic analysis revealed a complex karyotype comprising gain of chromosome segments 1q and loss of chromosome 4q (Figure 4). Fluorescence in situ hybridization (FISH) studies demonstrated a t(4;14) (q31;q32) - immunoglobulin heavy chain (IgH) translocation in 70% of the cells. The final diagnosis was anaplastic plasma cell myeloma with CD38 negativity.

## Discussion

Anaplastic myeloma is a morphological variant with a cluster of differentiation, genetic cytologic abnormalities, and poor prognosis. We encountered a 46-year-old female patient who had a nasal tumor, and the pathology report revealed that she had nasopharyngeal cancer. Chemotherapy and radiation therapy were used to treat the patient. After further investigation, the patient had many nosebleeds, including bone marrow aspiration and flow cytometry. The ultimate diagnosis was anaplastic plasma cell myeloma with CD38 negativity. After chemotherapy for a nasal tumor, the CD38 marker may become negative. These chemicals may have caused B-cell surface antigen damage, IgG disruption, similar to the case report of Patiño-Escobar B [3].

There are a few noteworthy aspects of this case: Firstly, anaplastic plasma cell myeloma is commonly associated with 17p(p53) deletion, 1q21 amplification, t(4;14). The presence of t(4;14) is defined as a cytogenetically high-risk disease by the International Myeloma Working Group (IMWG) [4]. Furthermore, while the IMWG does not officially classify FISH gain of 1q as high-risk, multiple studies have found it to be a poor prognostic sign, even when patients are treated with newer active agents [5,6]. There was a complex karyotype lesion in this patient, including a gain of 1q, a loss of 4q, and t(4;14) (q31;q32) - IGH translocation, which was associated with a poor prognosis.

Secondly, the current loss of CD38 has only been reported in some cases of anaplastic myeloma. Bataille et al. found that four new cases were diagnosed out of 29 CD38-negative multiple myelomas even after treatment. In contrast, in his other study, only two cases among 1000 patients were CD38 negative, either at the time of diagnosis or during relapse [7]. In addition, the association between anaplastic myeloma and negative CD38 has previously been reported in some cases in India and Arabia [8,9]. Moreover, Ichikawa has recorded a case of anaplastic plasma cells with CD38-dim and CD138-negative [2]. Loss of expression of target molecules on tumor cells is an important resistance mechanism to antibody-based therapies. This patient was CD38-negative at diagnosis and is therefore not a candidate for anti-CD38 therapy. The rare presentation in the first disease poses a challenge for aggressive diagnosis and treatment planning.

Thirdly, most plasma cells in multiple myeloma will be strongly positive for CD38, weakly positive for CD56, CD19 marker. In contrast, according to M Ocqueteau, a small group of patients with multiple myeloma has expressed the opposite immunological markers such as weak or negative CD38, strongly positive CD56, and negative CD19 [10].

## Conclusions

The pleomorphic morphology of anaplastic plasma cell myeloma can present a diagnostic dilemma. The combination of full panel immunophenotyping, including both CD38 and CD138 markers, with imaging techniques is required to establish an accurate diagnosis.
